# Corrigendum to Takagi et al. J Cachexia Sarcopenia Muscle, 13, 287‐295. https://doi.org/10.1002/jcsm.12906

**DOI:** 10.1002/jcsm.13172

**Published:** 2023-01-25

In Table 3, the details under the ‘Variable’ column are incorrect. Under the section ‘Sarcopenia’, ‘with sarcopenia group (*n* = 57)’ and ‘without sarcopenia group (*n* = 57)’ should have been swapped.

The correct details are highlighted in red as shown in the updated Table 3 below:

**TABLE 3:** Association of carnitine levels with sarcopenia, nutrition status, surgical outcome

Variable	TC	FC	AC
Sarcopenia	*P* = 0.0124	*P* = 0.0243	*P* = 0.102
Without sarcopenia group ( *n* = 57)	63.3 (29–102.5)	52.2 (24.8–93.5)	11 (4.2–24.5)
With sarcopenia group (*n* = 57)	56.6 (25.5–91.2)	46.8 (20.6–77.2)	9.8 (3.9–18.9)
Nutrition status^a^	*P* = 0.0003	*P* = 0.0001	*P* = 0.484
Low PNI group (*n* = 66)	54.3 (25.5–90.8)	44.2 (20.6–69.5)	10.2 (3.9–20.4)
High PNI group (*n* = 48)	64 (38–102.5)	53.4 (29.4–93.5)	10.7 (4.7–19.1)
Surgical outcome	*P* = 0.997	*P* = 0.872	*P* = 0.411
With postoperative complication (*n* = 37)	59.9 (32–90.8)	49.2 (22.8–77.2)	10.9 (5.4–24.5)
Without postoperative complication (*n* = 77)	59.9 (25.5–102.5)	49.6 (20.6–93.5)	10.2 (3.9–20.4)

AC, acylcarnitine FC, free carnitine; PNI, prognostic nutritional index; TC, total carnitine.

^a^ Cut‐off value is mean PNI value.

